# Overview of Phase-Change Electrical Probe Memory

**DOI:** 10.3390/nano8100772

**Published:** 2018-09-29

**Authors:** Lei Wang, Wang Ren, Jing Wen, Bangshu Xiong

**Affiliations:** 1 School of Information Engineering, Nanchang Hang Kong University, Nanchang 330069, China; wenj@nchu.edu.cn (J.W.); 42021@nchu.edu.cn (B.X.); 2 Shanghai Aerospace Electronic Technology Institute, Minxing district, Shanghai 201108, China; renwang23@126.com

**Keywords:** probe, phase-change, capping, bottom, optimization

## Abstract

Phase-change electrical probe memory has recently attained considerable attention owing to its profound potential for next-generation mass and archival storage devices. To encourage more talented researchers to enter this field and thereby advance this technology, this paper first introduces approaches to induce the phase transformation of chalcogenide alloy by probe tip, considered as the root of phase-change electrical probe memory. Subsequently the design rule of an optimized architecture of phase-change electrical probe memory is proposed based on a previously developed electrothermal and phase kinetic model, followed by a summary of the state-of-the-art phase-change electrical probe memory and an outlook for its future prospects.

## 1. Introduction

“Memory” is familiar to everyone owing to its data storage function in the long history of human life, evolving from its earliest forms such as cave wall, bone, and clay tablets, through its transitional forms including paper, punch card, and floppy disk, finally towards its present forms represented by hard disk, optical disc, and a portable Universal serial bus (USB) stick. Triggered by the pervasiveness of today’s digital services, “memory” is targeted to confine digital data in a nanoscale region within an ultrashort time scale at the cost of ultralow energy consumption. As a result, conventional digital data memories designed for mass/archival storage such as hard disks, tape, and optical discs cannot satisfy the aforementioned storage requirements because of their respective physical limits [1,2,3], thus leading to the debut of some emerging memories including, resistive random access memory (RRAM) [4], DNA memory [5], holographic memory [6], and probe memory [7,8,9,10,11]. In contrast to the first two infant technologies, probe memory that uses a nanoscale probe to change either the topography or physical properties of a storage medium has been subjected to intensive research during the last two decades owing to its technological maturity and fairly simple manufacturing process. Among the reported forms of probe memory, phase-change electrical probe memory has gained much more attention than its compatriots such as thermomechanical probe memory and magnetic probe memory owing to the advantageous traits of its storage medium: phase-change materials (PCMs). 

**Figure 1:**
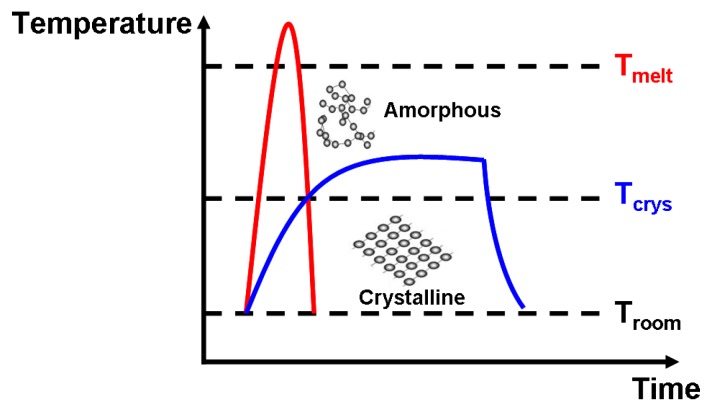
Thermal switching of PCMs between crystalline and amorphous phases.

PCMs that are widely adopted for phase-change electrical probe memory mainly fall into the category of the chalcogenide family (Group-16 elements, mainly S, Se, and Te) that can exist in both amorphous and crystalline phases with distinct electrical/optical properties. Amorphous chalcogenide that lacks an atomic long-range order can be transformed into its crystalline form by heating itself above the glass transition temperature and subsequently experiencing a slow cooling process, while heating crystalline PCMs with an atomic long-range order above the melting temperature and rapidly quenching them below room temperature can switch them back to the amorphous phase, as illustrated in Figure 1. Therefore, the storage functions of phase-change electrical probe memory mainly rely on such a reversible transition between the crystalline and amorphous states of PCMs. The write operation is realized by applying an electric stimulus via a conductive probe into the PCMs to heat them to the required temperature for phase transformation by the resulting Joule heating, whereas a relatively low electrical potential (to avoid erasure of the detected data) is applied to PCMs during the replay operation to distinguish the prewritten data from its background based on sensing current variations. 

**Figure 2:**
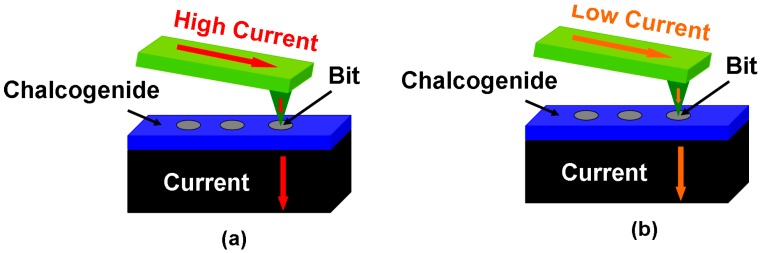
The electrical probe memory using Chalcogenide media when operated in (**a**) write mode and (**b**) readout mode.

Such a scheme is clearly depicted in Figure 2. Various attractive features of PCMs as a storage media such as great scalability (down to 5 nm) [12], rapid switching between two different phases (down to ns) [13], excellent stability at room temperature [14], and vast differences between certain physical properties [15] in different phases have been previously demonstrated, thus endowing phase-change probe memory with multiple merits such as ultrahigh capacity, fast write/read speed, long retention, and high readout contrast. Additionally, different from other probe memories that have to use physically sharp tips to sustain high storage capacity, the tip of the phase-change electrical probe memory is only required to be electrically sharp to restrict the write/read current in a desired conductive region for ultrahigh capacity. Thanks to this, the physical size of the tip can be enlarged by encapsulating its conductive core with certain insulated materials to reduce the pressure from which the tip suffers, and thus alleviate tip wear without affecting the resulting storage capacity. Because of these intriguing characteristics, phase-change electrical probe memory has been considered one of the most promising storage devices for the next-generation mass/archival storage industry and is worth particular attention to it. In this paper, different methods to switch the electrical or structural properties of PCMs using a nanoscale probe are first introduced, based on which the concept of a phase-change electrical probe memory is proposed. Subsequently the architecture and design rules of the phase-change electrical probe memory are elucidated, followed by a brief introduction of several already established device stacks by worldwide research groups as well as their respective write/readout performances. The weaknesses of the phase-change electrical probe memory that need to be addressed in conjunction with its future prospects are finally discussed.

## 2. Storage Mechanisms Review of Phase-Change Electrical Probe Memory

The storage function of phase-change electrical probe memory undoubtedly depends on the successful property transformation of the PCMs induced by an electrical probe. The complete study on the recording, reading, and erasing performances of the PCMs using an atomic force microscope (AFM) began in 1997 [16] when Kado et al. used a Si_3_N_4_ pyramidal probe coated with Cr and Au that underwent various electrical pulses between 2 V and 10 V to generate a series of written regions on an as-deposited amorphous GeSb_2_Te_4_ film that was deposited on a Pt bottom electrode. Although there is no pronounced difference in the topography image before and after the writing process, the electrical conductance of the written region is about two orders of magnitude higher than that of the unwritten region, detected by applying a bias voltage of ~0.5 V to the probe and plotting the conductance images. This clearly implies a successful electrical transformation in the amorphous film. The erasing operation was performed by injecting a negative bias voltage into the prewritten region to switch its high electrical conductance back to the original low state. As no marked topographical change was observed, Kado et al. attributed such a reversible transition of the electrical conductance to a Schottky-barrier tunneling mechanism in which electrons and holes injected from the cathode (probe) and anode (substrate) when subjected to a negative bias neutralize the space charges respectively and the surface energy barrier structure reverts to its original state. 

It is necessary to point out that the aforementioned recording/erasing operations reported by Kado did not involve any structural changes (i.e., phase transformations) and the electrically transformed region in this case is not very stable. To mitigate this situation, Tanaka et al. used a higher voltage pulse to generate conductance changes on amorphous GeSb_2_Te_4_ film [17] deposited on a Pt bottom electrode. The stability of the transformed region when experiencing a low bias voltage was demonstrated, and the X-ray diffraction patterns of the GeSb_2_Te_4_ film after application of the writing pulse clearly revealed the presence of structural changes in the prewritten region, meaning that a crystalline mark was successfully recorded. Triggered by this finding, the feasibility of using AFM to induce phase transformation in the well-known Ge_2_Sb_2_Te_5_ (GST) film either from amorphous to crystalline states or vice versa was also demonstrated by Tanaka et al. [18,19]. It should be noted that owing to the susceptibility of the crystalline GST to oxidation, the GST film during amorphization was covered by inactive liquid (3M, Fluorinert FC-43) to suppress oxidation. Another strategy to realize the storage function of the GST media through AFM is to combine an actual amorphous-crystalline phase transformation with a polarity-dependent resistance change, as proposed by Pandian et al. [20]. Based on their work, nanocrystalline marks were first recorded in the as-deposited amorphous GST film, and its resistance could be switched reversibly between an ON state and an OFF state by varying the polarity of the applied bias in order to accomplish the storage. Pandian et al. ascribed such a peculiar character to the solid-state electrolytic behavior of the chalcogenide material in which a conductive filament appears between two electrodes within the electrolyte media when exposed to a sufficiently strong electric field. Such a filament suffers a rupture when the applied electric field is reversed. Such a conjecture was most recently demonstrated by Sun et al. [21], who swept the bias voltage between positive and negative polarities to generate an I-V curve of a GST/Cr stack that exhibits typical bipolar switching characteristics with a pinched hysteresis loop cycling between a low-resistance state probably resulting from the partial crystallization of GST with an atomic migration of ions forming a conductive bridge, and a high resistance state stemming from the initially amorphous state. In addition to the Ge-Sb-Te media, the phase transformation of chalcogenide alloy induced by AFM was also achieved on GeTe_6_ media deposited on a SiO_2_ substrate with Ag contact [22], resulting in a low power threshold switching with an extremely low steady-state current in an ON state of 6–8 nA. Table 1 summarizes the physical mechanisms of the aforementioned approaches for electrically switching PCMs as well as their respective architecture and resulting mark sizes.

## 3. Design Approaches Review of Phase-Change Electrical Probe Memory

**Figure 3:**
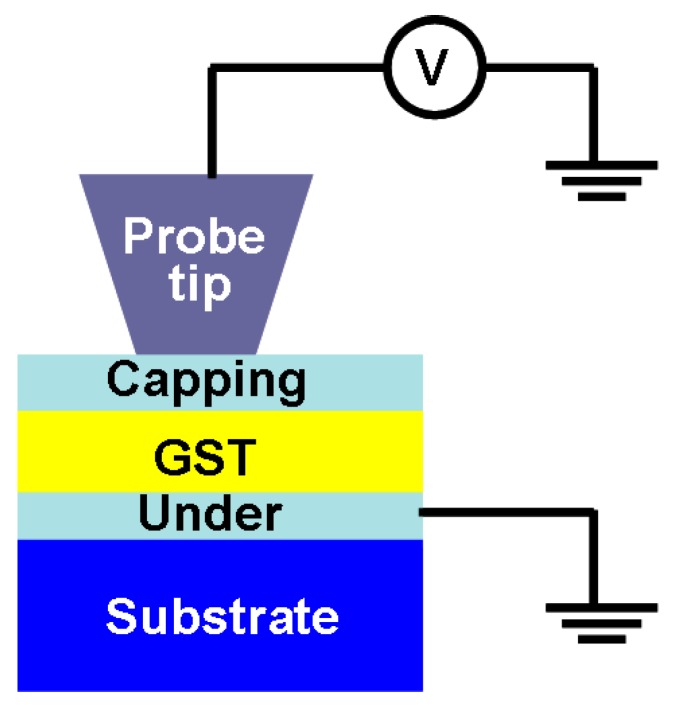
Schematic of phase-change electrical probe device.

Although the device architectures introduced above can readily enable the storage function that depends on either structural changes or resistance changes, it is not possible to directly reproduce them for practical applications owing to the vulnerability of the phase-change film to oxidation. As a result, a typical phase-change electrical probe memory composed of an AFM probe and a trilayer stack consists of a chalcogenide layer sandwiched by a capping layer and a bottom electrode deposited on a substrate, as illustrated in Figure 3.

As main components of the phase-change electrical probe memory, the geometrical and electro-thermal properties of the probe tip and the media stack may have a strong influence on the temperature distribution and the phase transformation extent inside the storage layer, and therefore needs to be determined carefully. To establish the roles of the probe tip and media stack on the physical performance of the phase-change electrical probe memory, and thus to optimize their design, a pseudo-3D model that consists of a Laplace equation, the heat conduction equation, and the Johnson-Mehl-Avrami-Kolmogorov (JMAK) equation was previously developed [7] to mimic the electrical process, the thermal process, and the phase-transformation process of the phase-change electrical probe memory, respectively. These are given by

(1) ∇⋅(σ⋅∇V)=0,

(2) $$\rho C_{p}\frac{\partial T}{\partial t}-k\cdot \nabla^{2}T=\sigma {\left|E\right|}^{2},$$

(3) $$\chi =1-\exp(-[\sum_{t}K_{JMAK}\cdot t{]}^{n}),$$

where *σ* is the electrical conductivity, *V* is the electric potential, *ρ* is the density, *C_P_* is the heat capacity, *T* is the temperature, *k* is the thermal conductivity, *E* is the electric field, *χ* is the crystal fraction, *K_JMAK_* is the crystallization rate, *t* is the time, and *n* is the Avrami factor. As the physical reality of this developed model was already demonstrated in previous literature [7] and its details were also introduced elsewhere, it is directly implemented here to assess the impact of the probe tip and media stack on the device performance and to finalize the design rule of the phase-change electrical probe memory. It should also be noticed that since such a model only applies to a storage device based on a phase transformation (i.e., structural change) mechanism, the design criterion of phase-change probe memory relying on resistance changes will not be discussed in this paper. 

### 3.1. Role of Probe Tip

**Figure 4:**
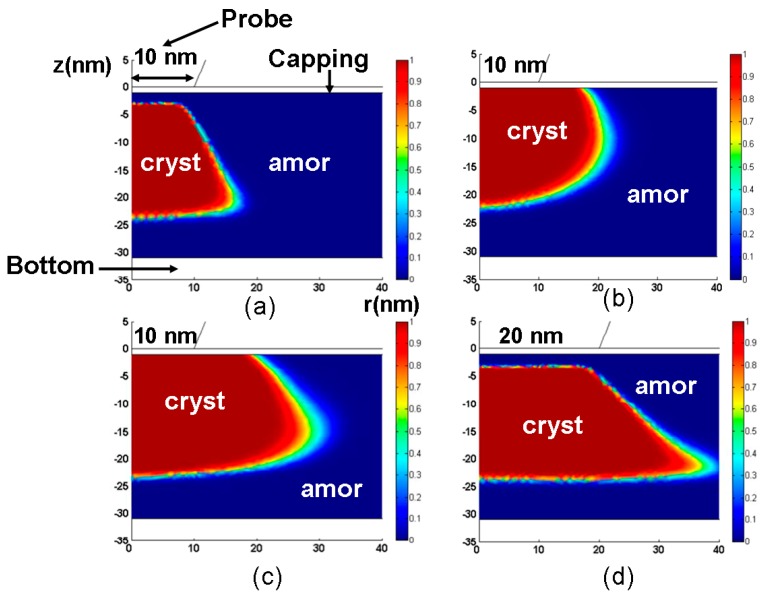
The resultant crystalline bits using a probe tip with (**a**) a 10 nm radius, an electrical conductivity of 104 Ω^−1^⋅m^−1^, and a thermal conductivity of 100 W⋅m^−1^⋅K^−1^. (**b**) a 10 nm radius, an electrical conductivity of 10^7^ Ω^−1^⋅m^−1^, and a thermal conductivity of 100 W⋅m^−1^⋅K^−1^. (**c**) a 10 nm radius, an electrical conductivity of 10^4^ Ω^−1^⋅m^−1^, and a thermal conductivity of 2 W⋅m^−1^⋅K^−1^. (**d**) a 20 nm radius, an electrical conductivity of 10^4^ Ω^−1^⋅m^−1^, and a thermal conductivity of 100 W⋅m^−1^⋅K^−1^.

The influence of the geometrical (tip diameter here) and electrothermal properties of the probe tip on the consequent phase-change behavior of the chalcogenide (GST alloy here) layer is schematically shown in Figure 4 by varying the electrical conductivity, thermal conductivity, and diameter of the probe tip from 10^4^ Ω^−1^⋅m^−1^ to 10^7^ Ω^−1^⋅m^−1^, 100 W⋅m^−1^⋅K^−1^ to 2 W⋅m^−1^⋅K^−1^, and 10 nm to 40 nm respectively. A comparison between Figure 4a,b clearly reveals that using a probe tip with a higher electrical conductivity results in a larger crystalline mark than with a lower electrical conductivity for a given electrical pulse. This is expected, as the use of a highly electrically conductive probe tip can effectively lower the entire device resistance and thus enable larger current and more Joule heating inside the storage layer. The situation in which a probe tip with a higher electrical conductivity can generate a larger mark also implies that the same mark size as a case with a less electrically conductive probe tip can be secured using a smaller electric pulse, thereby leading to lower energy consumption. The role of the thermal conductivity of the probe tip on the resulting mark is opposite to the electrical conductivity case, since using a probe tip with a smaller thermal conductivity forms a larger crystalline mark than that with higher thermal conductivity for a given electric pulse. This can be easily understood as follows: the heat dissipation toward the surrounding air can be heavily suppressed owing to the low thermal conductivity of the probe tip, and more Joule heating is accumulated inside the storage layer to enable larger marks. In this case, it is possible to use lower electric pulse and less thermally conductive probe tip to generate the same mark size as the case with a larger thermal conductivity. In addition to its electro-thermal properties, the size of the probe tip (i.e., diameter) is also a key factor in the phase-transformation of the storage layer, as reflected in Figure 4d. In comparison with Figure 4a, Figure 4d clearly indicates an important finding in that the minimum size of the phase-transformed region for probe storage is mainly determined by the diameter of the probe tip. As a result, a probe tip with a smaller diameter is always desired in order to enable ultra-high recording density.

**Figure 5:**
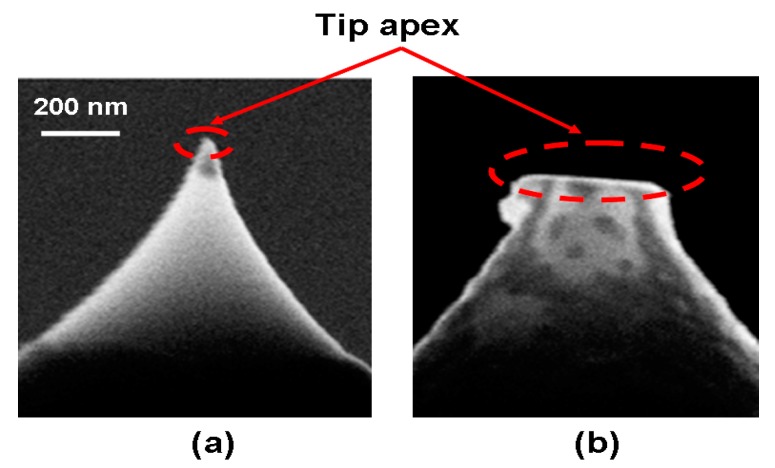
SEM images of a Si tip (**a**) without wear and (**b**) with wear.

According to the results presented so far, the electrical and thermal conductivities of the probe tip contribute to the energy consumption of the resulting phase transformation, while the size of the probe tip governs the maximum recording density of the phase-change electrical probe memory. Thanks to this, an optimized design of the probe tip should have high electrical conductivity, low thermal conductivity, and a short diameter. As introduced in Section 2, a conventional Si probe tip coated with some highly electrically conductive metal has been commonly used for probe storage. Although such a probe tip is able to provide high write/read current for a small electric pulse, its large thermal conductivity would deteriorate the heat dissipation and causes more Joule heating to escape from the probe tip. Moreover, repeated scanning of the probe tip over the surface of the sample exacerbates the tip wear. This equivalently increases the diameter of the tip and reduces the resulting density, as illustrated in Figure 5.

**Figure 6:**
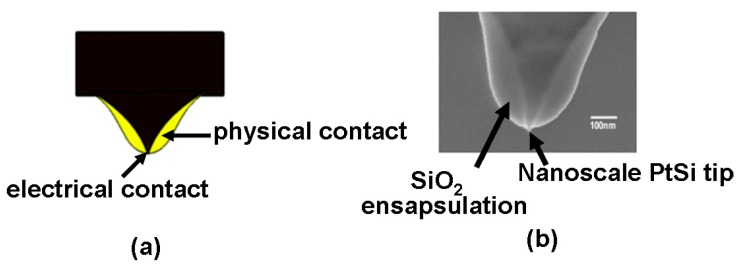
(**a**) Schematic of the ‘encapsulated’ tip concept, and (**b**) the experimentally fabricated encapsulated tip. Reproduced with permission from [24]. Copyright IOP Science, 2009.

To improve upon this, a novel probe tip prototype that employs a SiO_2_ encapsulated Si tip with PtSi at the tip apex was previously proposed [23,24] and physically fabricated, as shown in Figure 6. Accounting for its great antiwear character and high electrical conductivity, forming PtSi at the tip apex can significantly mitigate the tip wear issue and strengthen the conduction performance of the probe tip. More importantly, the use of dielectric encapsulation (SiO_2_) can increase the physical diameter of the tip and thus contribute to lower levels of wear owing to the reduced pressure between the tip and sample. Therefore, combining PtSi at the tip apex with SiO_2_ encapsulation would simultaneously not only provides for a small electrical contact diameter that leads to an ultra-high write and read density, and also exhibits a large physical contact diameter to mitigate tip wear. Owing to the above reasons, this probe tip has been extensively used in both practical experiments and theoretical simulations related to probe storage applications.

### 3.2. Role of Capping Layer

**Figure 7:**
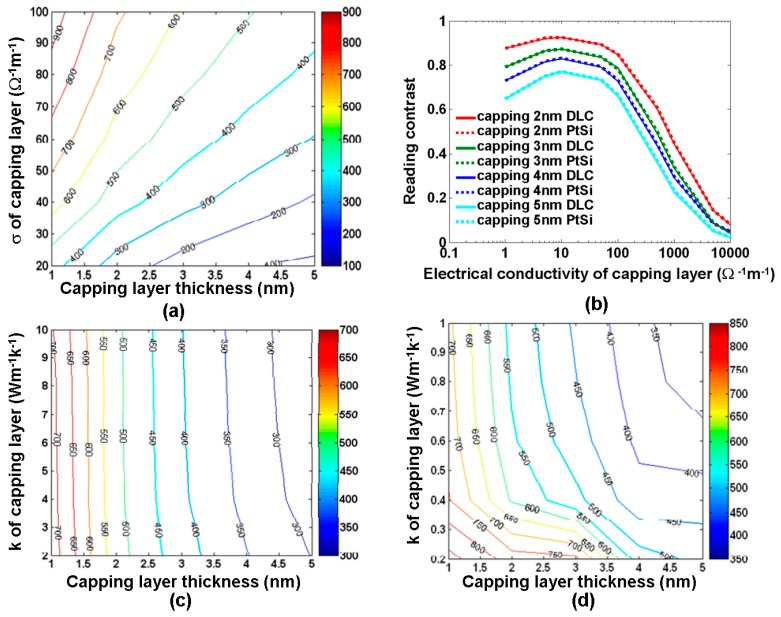
(**a**) maximum temperature (°C) inside the GST layer during crystallization as a function of the electrical conductivity and the thickness of the capping layer by a 8 V pulse of 100 ns. (**b**) reading contrast as a function of the electrical conductivity and the thickness of the capping layer for a single crystalline bit by a 1 V potential. (**c**,**d**) maximum temperature inside the GST layer during crystallization as a function of the thermal conductivity and the thickness of the capping layer by a 8 V pulse of 100 ns. *σ* and *k* represent the electrical and thermal conductivities of capping layer respectively. Reproduced with permission from [26]. Copyright Ingenta Connect, 2015; [27]. Copyright Springer, 2014; [28]. Copyright Iop Science, 2014.

A major difference between the phase-change electrical probe memory and media stacks described in Section 2 is that the phase-change electrical probe memory needs a capping layer to protect the storage media from oxidation and wear. Therefore, the materials used for the capping layer are generally required to have high hardness and great stability. Additionally, as a conductive bridge to connect the probe tip to the storage layer, its layered thickness and electrothermal properties drastically affect the device resistance and the resulting current density that liaises directly with the final extent of the phase transformation. Taking the above factors, diamond-like carbon (DLC) films previously used as protective coatings in objects such as magnetic storage disks, car parts, and optical windows, has been considered as one of the most appropriate materials for the capping layer [25], owing to its high mechanical hardness, chemical inertness, and optical transparency. To induce the required phase transformation without causing extra energy consumption, the aforementioned electrothermal model was employed previously to find out the optimized layered thickness and the electrothermal properties of the DLC capping layer [26,27,28]. This is shown in Figure 7.

As clearly shown in Figure 7a, the maximum temperature inside the storage layer (GST alloy here) during the crystallization process varies from 100 °C to 900 °C when the electrical conductivity and the thickness of the DLC capping layer changes from 1 nm to 5 nm and from 20 Ω^−1^⋅m^−1^ to 100 Ω^−1^⋅m^−1^, respectively. It was found that for a given electrical conductivity, using a thinner capping layer induces a higher temperature inside the storage layer than a thicker capping layer. This is because a capping layer with a smaller thickness enables a lower device resistance and thus enhances the current density and the resulting Joule heating inside the storage layer for a given write pulse. On the other hand, a capping layer with a higher electrical conductivity results in higher temperature than that a lower electrical conductivity for a fixed layer thickness, also owing to a decrease in the device resistance. According to Figure 7a, the crystallization temperature within a nanosecond regime (~400 °C) can be easily achieved using either a low electrical conductivity with a thin thickness or a thick thickness with a high electrical conductivity. Please note that in addition to the writing process, the choices for the electrical conductivity and thickness of the capping layer may also affect the sensing current during the readout process as well as the reading contrast defined by I_max_ − I_min_/I_max_ + I_min_, where I_max_ and I_min_ are the maximum and minimum readout current during the readout process. This is shown in Figure 7b. As observed from Figure 7b, the reading contrast is increased by reducing the thickness of the capping layer, since this decreases the spreading resistance, thus allowing for a higher readout current signal. Owing to the above reason, a maximum reading contrast of ~0.92 is acquired by a 2 nm capping layer with an electrical conductivity of 10 Ω^−1^⋅m^−1^. The reading contrast as a function of the electrical conductivity of the capping layer presents an intriguing trend because it first increases along with the capping layer electrical conductivity up to 10 Ω^−1^⋅m^−1^, followed by a gradual decline by further increasing its electrical conductivity to 10,000 Ω^−1^⋅m^−1^. The initial increase of the reading contrast stems from the use of a capping layer with higher electrical conductivity that leads to a larger sensing current, whereas further increasing the electrical conductivity of the capping layer intensifies the current spreading effect. In this case, the difference between I_max_ and I_min_ is less pronounced, thus lowering the reading contrast. The dependence of the maximum temperature inside the storage layer during crystallization on the thermal conductivity of the DLC capping is illustrated in Figure 7c,d by varying the thermal conductivity of DLC capping from 0.2 W⋅m^−1^⋅K^−1^ to 10 W⋅m^−1^⋅K^−1^. As seen in Figure 7c,d, for a fixed layer thickness, DLC capping with a thermal conductivity greater than 1 W⋅m^−1^⋅K^−1^ has a slight impact on the resulting maximum temperature inside the storage layer. This is expected because within this range the thermal conductivity of the DLC capping is much larger than that of the storage layer (0.2 W⋅m^−1^⋅K^−1^ in the amorphous phase and 0.58 W⋅m^−1^⋅K^−1^ in the crystalline phase) and can barely affect the heat dissipation through itself. However, when restricting the thermal conductivity of DLC capping within 1 W⋅m^−1^⋅K^−1^, it was observed that using DLC capping with a smaller thermal conductivity can greatly augment the maximum temperature inside the storage layer. In this case, the thermal conductivity of the DLC capping is comparable to that of the storage layer. In addition, using DLC capping with a smaller thermal conductivity can substantially suppress the heat dissipation through the capping layer itself and thus increase the temperature inside the storage layer.

**Figure 8:**
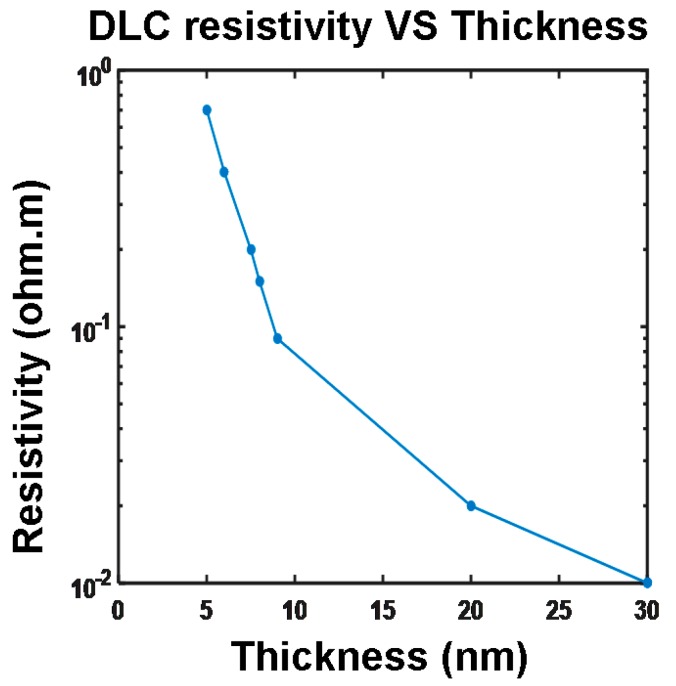
The dependence of the DLC film thickness on its electrical resistivity. Reprinted with permission from [29]. Copyright De Gruyter, 2016.

Based on the above analysis, a DLC capping film with a thin thickness, intermediately high electrical conductivity, and low thermal conductivity is desired in order to achieve the required phase-transformation temperature and a high reading contrast within the least energy consumption. Thus, in most studies, an optimized capping layer was designed as a DLC film with a thickness of 2–5 nm, an electrical conductivity of 50–100 Ω^−1^⋅m^−1^, and a thermal conductivity of 0.2–0.5 W⋅m^−1^⋅K^−1^. However, it was recently reported that DLC thin films exhibits strong thickness-dependent electrical conductivity [29], as illustrated in Figure 8. 

According to Figure 8, the electrical conductivity of the DLC film slowly increases from 20 Ω^−1^⋅m^−1^ to 100 Ω^−1^⋅m^−1^ by varying its thickness from 5 nm to 30 nm, after which its electrical conductivity is almost independent of the film thickness. As a result, it seems that such an optimized capping layer designed from theoretical simulations cannot be attained experimentally, as it is not possible to fabricate a DLC film with sufficient thickness and electrical conductivity simultaneously. Moreover, the use of a DLC capping with low electrical conductivity introduces a large electrical contact resistance at the tip-capping interface, calculated by [30]:

(4) $$R_{contact}=\frac{\rho_{capping}+\rho_{tip}}{4{\left\{r^{2}-{\left[r-{\left(\frac{9F^{2}}{16rE^{*}{}^{2}}\right)}^{1/3}\right]}^{2}\right\}}^{1/2}},$$

where *ρ_capping_* and *ρ_tip_* are the resistivities of the capping layer and tip, respectively; *r* is the radius of the tip apex; *F* is the tip loading force, and E* is the effective Young’s modulus. According to Equation (4), the value of the contact resistance for a 5 nm thick DLC capping with an electrical conductivity of 20 Ω^−1^⋅m^−1^ is approximately 400 kΩ for a typical tip force of 300 nN and for ρ_tip_ = 0.3 × 10^−6^ Ω⋅m. Such a large contact resistance undoubtedly requires a much larger electric pulse to induce the required phase transformation, thus causing extra energy consumption. In this case, the practicality of using DLC film for the capping layer of the phase-change electrical probe memory has recently been challenged. 

**Figure 9:**
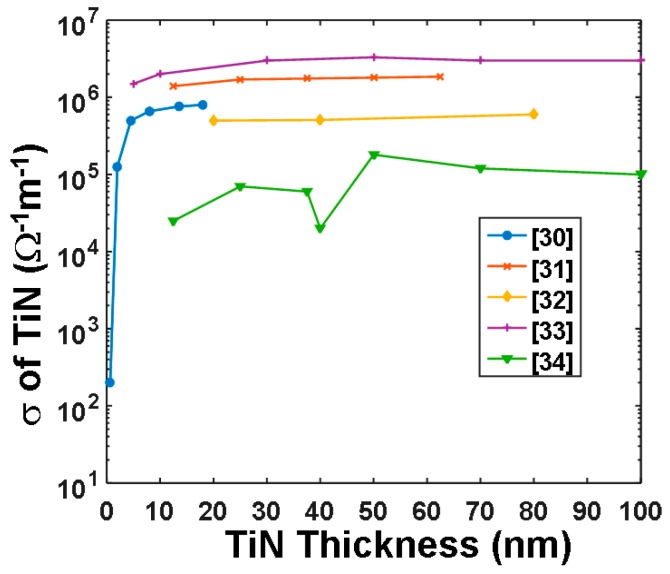
The electrical conductivity of TiN films with respect to its thickness for different deposition techniques.

In addition to DLC film, titanium nitride (TiN) film has most recently been considered as another prospective material for the capping layer of the phase-change electrical probe memory owing to its high electrical conductivity and intermediately low thermal conductivity. As clearly shown in Figure 9, depending on the specific deposition technique, the electrical conductivity of the TiN film varies between 10^5^ Ω^−1^⋅m^−1^ and 10^7^ Ω^−1^⋅m^−1^ when its thickness changes from 2 nm to 100 nm [31,32,33,34,35], and a 2 nm thick TiN film allows for an electrical conductivity up to 2 × 10^5^ Ω^−1^⋅m^−1^. This is obviously suggested in order to achieve a small interfacial contact resistance and possibly a low electric pulse for the required phase transformation.

**Figure 10:**
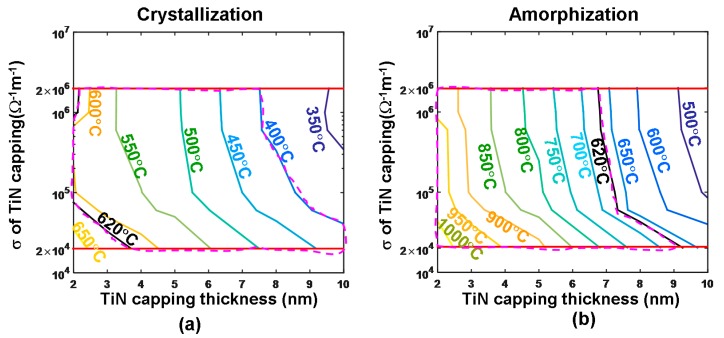
Maximum temperature inside the GST layer as a function of the electrical conductivities and the thickness of the TiN capping layer for (**a**) crystallization by a 5.5 V of 180 ns and (**b**) amorphization by a 6.5 V of 150 ns. The investigated electrical conductivity range of the TiN capping layer is bounded by two red lines, while the resulting optimized regions are encompassed by the violet dashes. Reprinted with permission from [36]. Copyright Springer, 2018.

To prove this, some preliminary studies were performed to assess the role of the layered thickness and electrothermal properties of TiN films on the writing performance of the phase-change electrical probe memory [36], as shown in Figure 10. 

According to a comparison between Figure 7 and Figure 10, it was found that the use of a thin TiN capping layer enables a larger temperature inside the storage layer than a thick TiN layer, owing to a decrease in the entire device resistance. Nevertheless, for a fixed layer thickness, the maximum temperature inside the storage layer is almost independent of the electrical conductivity of the TiN capping, since the TiN capping plays a minor role in determining the entire device resistance owing to its ultrahigh electrical conductivity. Therefore, in order to reach both crystalline and amorphous temperature (~620 °C) using one media stack, the layered thickness and the electrothermal properties of the TiN capping are optimized to be 2 nm, 2 × 10^5^ Ω^−1^⋅m^−1^, and 12 W⋅m^−1^⋅K^−1^, respectively, clearly matching the experimental measurements given in Figure 9. The capability of a phase-change electrical probe memory with the proposed TiN capping to achieve an ultra-high recording density was already demonstrated, while its feasibility of achieving high reading contrast remains questionable and needs further investigation. 

### 3.3. Role of the Storage Layer

**Figure 11:**
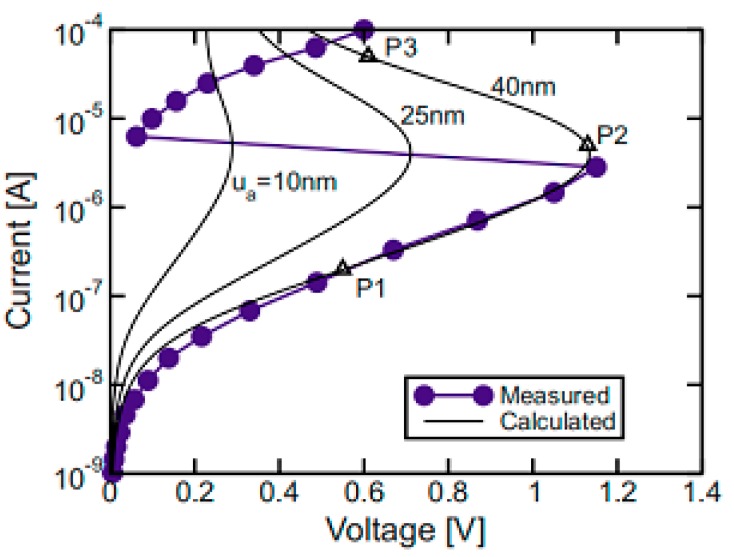
Threshold voltages for a phase-change memory cell with amorphous GST for three values of the thickness, namely u_a_ = 10, 25, and 40 nm. Reprinted with permission from [37]. Copyright APS Physics, 2008.

It is well known that the current passing through the chalcogenide layer undergoes a drastic increase once the voltage across the layer exceeds a critical value. This is known as the “threshold switching” phenomenon. Such a critical voltage is usually named a threshold voltage, which can be simply described as a product of the chalcogenide layer thickness and the threshold field that is generally considered as a constant. Therefore, a thin chalcogenide layer is always anticipated in order to reduce the threshold voltage as well as the resulting energy consumption [37], as schematically verified in Figure 11. 

**Figure 12:**
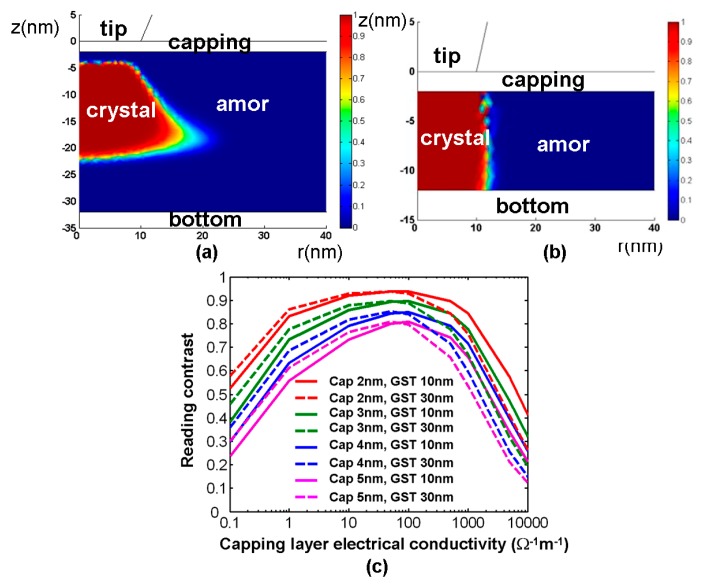
The resulting crystalline bit from a GST layer with a thickness of (**a**) 30 nm and (**b**) 10 nm, and (**c**) the resulting reading contrast as a function of the GST layer thickness for a single crystalline bit. Reproduced with permission from [7]. Copyright Ieee Xplore, 2006; [27]. Copyright Springer, 2014; [37]. Copyright IOP Science, 2014.

According to Figure 11, changing the GST storage layer thickness from 10 nm to 40 nm results in a variation of the threshold voltage from 0.3 V to 1.2 V, indicating that the use of a thin GST layer allows for higher current density by a smaller threshold voltage than the thick GST layer. Additionally, the influence of the storage layer (GST in this case) thickness on the write/read performances of the phase-change electrical probe memory has also been evaluated previously [7,28,38], as illustrated in Figure 12. A comparison between Figure 12a,b clearly shows that a 30 nm thick GST layer results in a trapezoidal-shaped crystalline bit with untransformed amorphous regions on its top and bottom, while reducing the GST layer thickness to 10 nm enables a perfectly cylindrical crystalline bit that extends through the entire storage layer. Such a shape difference may arise from a conjecture that a GST layer with larger thickness attenuates the heat penetration effect and thus induces insufficient heating at the interfacial regions (i.e., tip-capping interface and GST-bottom interface). It is evident that the crystalline bit shown in Figure 12a severely degrades the reading signal owing to its surrounding amorphous region. In terms of the reading contrast, it is denoted in Figure 12c that the reading contrast resulting from the 30 nm case almost overlaps with the 10 nm case when the electrical conductivity of the capping layer is less than 50 Ω^−1^⋅m^−1^. Meanwhile the 10 nm GST layer exhibits a larger reading contrast than the 30 nm case with a capping layer having an electrical conductivity greater than 50 Ω^−1^⋅m^−1^. The initial coincidence between two cases maybe interpreted based on the large difference in resistance between the capping layer and the GST layer. In this case, the capping layer contributes to the majority of the device resistance, and thus the thickness of the GST layer has a slight impact on the resulting reading contrast. For the case of an electrical conductivity of the capping layer that is higher than 50 Ω^−1^⋅m^−1^, the entire device resistance is mainly dominated by the GST layer. In this case, the GST layer with 10 nm thickness induces a larger readout current than the 30 nm case, thereby leading to a higher reading contrast. Based on the results presented so far, an optimized GST layer is expected to have a thin thickness in order to provide a regular-shaped bit and a high reading contrast.

### 3.4. Role of the Bottom Layer

**Figure 13:**
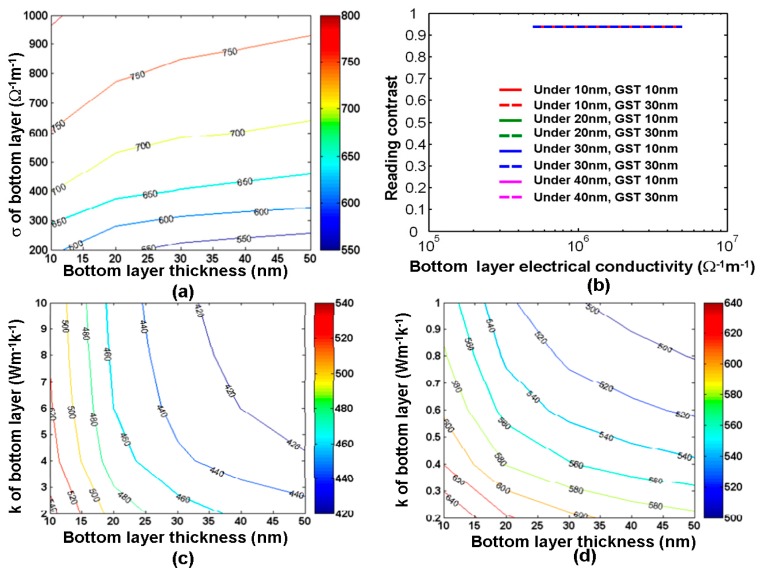
(**a**) maximum temperature (°C) inside the GST layer during crystallization as a function of the electrical conductivity and the thickness of the bottom layer by a 8 V pulse of 100 ns. (**b**) reading contrast as a function of the electrical conductivity and the thickness of the bottom layer for a single crystalline bit by a 1 V potential. (**c**,**d**) maximum temperature inside the GST layer during crystallization as a function of the thermal conductivity and the thickness of the bottom layer by a 8 V pulse of 100 ns. *σ* and *k* represent the electrical and thermal conductivities of capping layer respectively. Reproduced with permission from [26]. Copyright Ingenta Connect, 2015; [38]. Copyright Iop Science, 2014; [28]. Copyright Iop Science, 2014.

A bottom layer that serves mainly as the bottom electrode is usually implemented to collect the write/read current. Hence, the bottom layer needs a high electrical conductivity to sustain sufficiently high current density for a small electrical input, and a low thermal conductivity to guarantee sufficient heating to occur at the bottom of the GST layer. To follow this design rule, DLC film was considered as the most suitable media for the bottom electrode of the phase-change probe memory at the early stages, and its potential link to the maximum temperature inside the storage layer associated with the resulting reading contrast is clearly revealed in Figure 13 [26,28,38]. According to Figure 13a, the thickness and electrical conductivity of the bottom layer play a less important role in determining the maximum temperature inside the storage layer than the capping layer case. The maximum temperature varies from 550 °C to 750 °C within the range of the thickness and the electrical conductivity of the bottom layer from 10 nm to 50 nm and from 200 Ω^−1^⋅m^−1^ to 1000 Ω^−1^⋅m^−1^, respectively. This is probably because the resistance of the bottom layer is much lower than that of the capping layer and the storage layer, and thus cannot affect the resulting current density. Moreover, it is clearly indicated in Figure 13b that the resulting reading contrast is not significantly affected by the thickness and the electrical conductivity of the bottom layer. In this case, a thin bottom layer with a high electrical conductivity is desired to inducea sufficient temperature under a small external excitation without impairing the reading contrast. Compared with the capping layer, the thermal conductivity of the bottom layer also plays a less critical role in the maximum temperature inside the storage layer according to Figure 13c,d, as the maximum temperature varies from 400 °C to 640 °C within a range of the bottom layer thickness from 10 nm to 50 nm and thermal conductivity from 0.2 W⋅m^−1^⋅K^−1^ to 10 W⋅m^−1^⋅K^−1^. It was noticed that for a given layer thickness, using a bottom layer with a smaller thermal conductivity enables a higher temperature inside the storage layer than that with a larger thermal conductivity. This is because a bottom layer with a smaller thermal conductivity can effectively suppress the heat dissipation through the substrate and thus retain enough Joule heating for the required phase transformation. On the other hand, the maximum temperature inside the storage layer is reduced by increasing the thickness of the bottom layer for a given thermal conductivity. This is probably ascribed to the increase in the device resistance that in turn lowers the induced current density and consequent Joule heating. According to the above analysis, an optimized DLC bottom layer was previously proposed for a 10 nm thick film with an electrical conductivity of 100 Ω^−1^⋅m^−1^ and a thermal conductivity of 0.5 W⋅m^−1^⋅K^−1^. However, its experimental availability remains a challenge. 

**Figure 14:**
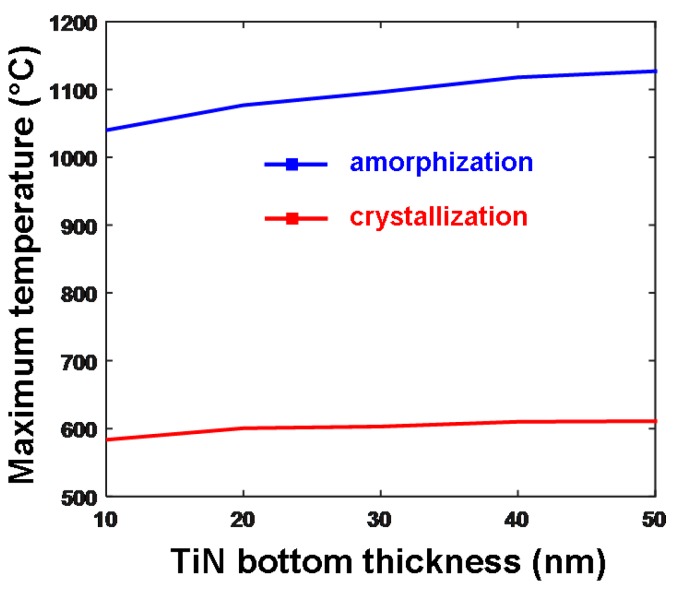
Maximum temperature (°C) as a function of the thickness of the TiN bottom layer during both crystallization and amorphization processes. Reproduced with permission from [36]. Copyright Springer, 2018.

In addition to the DLC layer, TiN film also suits the aforementioned design rule for an optimized bottom electrode owing to its ultra-high electrical conductivity, intermediate thermal conductivity, and practical availability. To prove this, one research group recently varied the electrical conductivity and the thickness of the TiN films to establish their influence on the resulting maximum temperature inside the storage layer for both crystallization and amorphization processes. This is shown in Figure 14. Please note that the electrical conductivities of the TiN films for different thicknesses in Figure 14 are directly extracted from Figure 9, and the thermal conductivity is set at 12 W⋅m^−1^⋅K^−1^. As illustrated in Figure 14, the maximum temperature inside the storage layer was slightly enhanced by increasing the thickness of the TiN bottom layer, which can be explained due to the further separation of the GST layer from the substrate that consequently reduces the heat dissipation. However, such a temperature variation is not as eminent as that in the capping layer. For this reason, an optimized TiN bottom layer is generally preferred to have a 40 nm thickness with an electrical conductivity of 2 × 10^6^ Ω^−1^⋅m^−1^ and thermal conductivity of 12 W⋅m^−1^⋅K^−1^.

According to above analysis, both capping and bottom layers need to be designed in such a way that the heat loss from the device is minimized. Since majority of the heat loss is caused through the capping and bottom electrodes, the capping and bottom layer materials with low thermal conductivities and larger thermal boundary resistance (TBR) are highly desired. However, as conventional electrode materials with low thermal conductivities such as C and Ti usually give rise to low TBR at nanoscale thickness, the materials chosen for both capping and bottom layers are generally required to have multiple compositions rather than a single constitute. The composition of the phase-change materials mainly lie on or in the vicinity of a pseudo binary line that joins the stoichiometric compounds: Sb_2_Te_3_ and GeTe, in the ternary phase diagram of Ge-Sb-Te. In search of faster materials, undoped and slightly Ge-doped Sb devices with a composition of Ge_15_Sb_85_ have been fabricated. It should be also noted that RRAM that adopts asymmetric electrodes has been recently reported to exhibit a polarity-dependent ‘RESET’ current [39]. It is natural to conceive whether such polarity dependence can be also found on phase-change electrical probe memory. It is likely that the polarity of the applied pulse has a minor impact on the resulting bit for the stacks that make use of symmetric electrode such as DLC/GST/DLC or TiN/GST/TiN. However, for the typical design such as TiN/GST/DLC, switching pulse polarity would have an evident change on the resulting current spreading effect and may affect the resulting bit size, which is worth further investigation in the future.

## 4. Current status of Phase-Change Electrical Probe Memory

### 4.1. Recent Progress on Phase-Change Electrical Probe Memory from Authors’ Group

**Figure 15:**
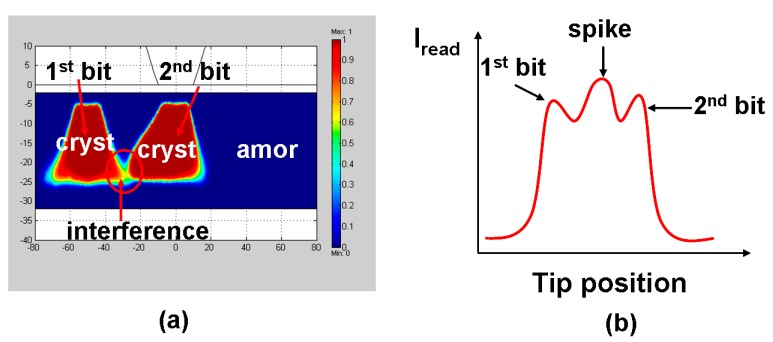
(**a**) Thermal cross-talk effect occurring when writing two successive bit and (**b**) the resulting readout signal from (**a**).

It should be pointed out that the aforementioned optimized design is based on a pseudo-3D model (i.e., 2D cylindrical symmetry) that is however incapable of mimicking the writing of multiple bits, as in this case the cylindrical symmetry trait will be destroyed. As a result, in order to more closely imitate the practical setup, a pure 3D physically realistic model is essentially required to simulate the writing of multiple bits as well as the resulting thermal-cross talk effect defined as the interference between the previously written bit and its adjacent bit being written due to the thermal diffusion effect, as depicted in Figure 15. It was found in Figure 15a that decreasing the separation between the two successive written bits would cause an appearance of a ‘tail’ on the bit being written (2nd bit) towards the previously written bit (1st bit). This is expected as the region where the 1st bit is formed exhibits lower electric resistance than the surrounding background and the write current during the write of the 2nd bit would preferably flow towards the 1st bit to form such a ‘tail’. The presence of this ‘tail’ would obviously generate a ‘spike’ signal during the readout of these two bits as shown in Figure 15b, and thus makes it hard to discern the written bit from the surrounding background.

**Figure 16:**
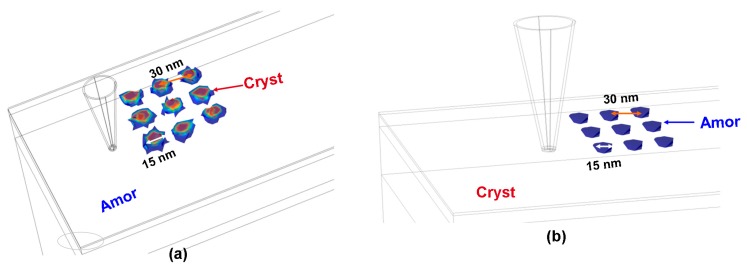
The generated 3 × 3 bit array using the developed 3D model with the optimized characteristic parameters in (**a**) crystalline and (**b**) amorphous phases.

To inhibit the thermal cross-talk effect without sacrificing the achievable density, we have most recently transformed the previously pseudo-2D model into pure 3D dimension, whereby the thermal cross-talk effect was investigated by calculating the crystal fraction and maximum temperature between two adjacent bits for crystallization and amorphization respectively by appropriately tailoring the geometrical and electro-thermal properties of layered structures. It was revealed through our findings that a thin capping layer with an intermediately high electrical conductivity and low thermal conductivity is preferred to suppress the thermal cross-talk effect between adjacent bits, while the bottom electrode plays a minor role in influencing the thermal cross-talk effect. In this case, the previously designed stack has been modified to a 10 nm GST layer sandwiched by a 5 nm DLC layer with an electrical conductivity of 50 Ω^−1^⋅m^−1^ and a thermal conductivity of 0.5 W⋅m^−1^⋅K^−1^, and a 40 nm TiN bottom with an electrical conductivity of 5 × 10^6^ Ω^−1^⋅m^−1^ and a thermal conductivity of 12 W⋅m^−1^⋅K^−1^ so as to minimize the thermal cross-talk effect to the utmost, as demonstrated in Figure 16.

**Figure 17:**
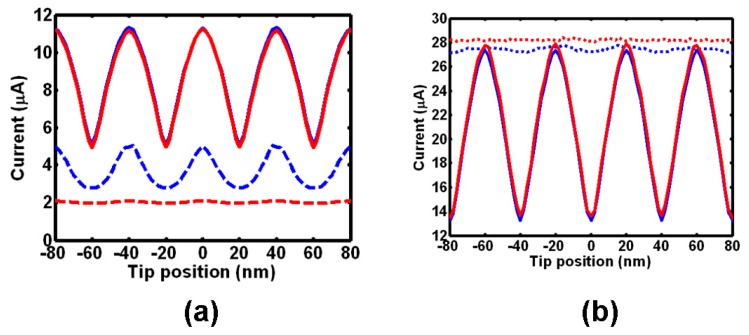
The resulting readout current for (**a**) multiple crystalline bit arrays, and (**b**) multiple amorphous bit arrays. The blue line and red line represent the readout signals for 40 nm track pitch and 60 nm track pitch respectively, while the solid line and dotted line indicate the ‘on-track’ scanning and ‘off-track’ scanning respectively.

In addition to the improvement of the write model, the necessity to have a pure 3D readout model to assess the readout of multiple bits associated with the resulting inter-symbol interference becomes of importance, giving rise to the revolution of the previously 2D readout model to its 3D form. In this newly developed 3D model, the shapes of the crystalline and amorphous bits are set to be cylindrical and semi-elliptical respectively to match the observations obtained from the previous 3D write model, and multiple bit arrays instead of a single bit are also introduced into this model to simulate the real readout environment, and to investigate the inter-track inferences between two adjacent bits, leading to Figure 17. As can be seen from Figure 17a, the resulting readout signals exhibit sinusoidal shape for both 40 nm and 60 nm track pitch; the maximum readout current (peak) is obtained when tip is on top of the crystalline bit because of its large electrical conductivity, while the minimum current (valley) is induced from reading the neighboring amorphous region between two adjacent bits. However, the case with 60 nm track pitch shows much smaller inter-track interference than that with 40 nm track pitch when tip scans over the ‘off’ track region. In this case, for reading crystalline bits from amorphous matrix, the readout signal will be most affected by adjacent tracks when the tip is reading on amorphous region and the adjacent track contains crystalline bits. The shape of the readout signal possessed from detecting amorphous bits from crystalline matrix, as clearly shown in Figure 17b, is found to be similar to the case of reading crystalline bits, as the current peak and valley correspond to the position of crystalline matrix and amorphous bit respectively. More importantly, there is no eminent difference witnessed on the inter-track interference between 40 nm and 60 nm track pitch, meaning that readout of amorphous bits from crystalline matrix can effectively overcome the inter-track interference without penalizing the recording density.

**Figure 18:**
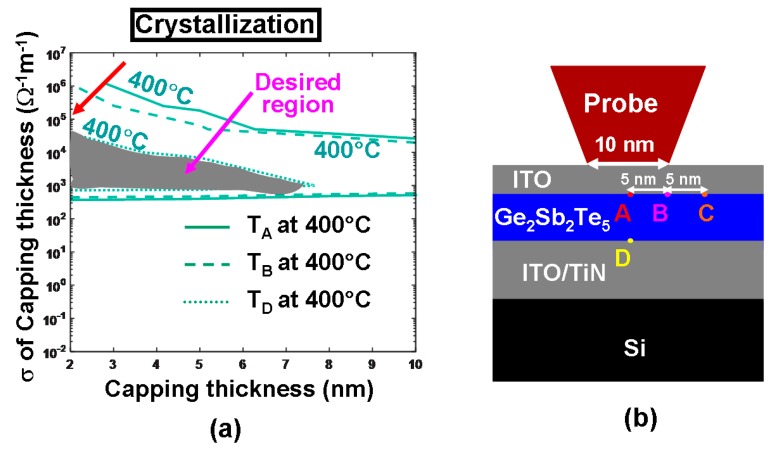
(**a**) Temperature contours at some pre-defined points (A, B, C, D) as a function of electrical conductivities (*σ*) and thickness of the ITO capping layer during crystallization and (**b**) locations of the pre-defined points.

It should be noticed that both the media stack design using DLC/GST/DLC or that with TiN/GST/TiN have their respective drawbacks, thus limiting their practical applications. The stack design with DLC capping usually causes a large contact resistance at tip-capping interface and thereby give rise to extra energy consumption, while for stack structure with TiN layer, the Ti ions may readily diffuse into the GST layer during SET/RESET operations to degrade the device endurance cycle [40]. As a result, a new type of probe memory architecture that has a GST layer sandwiched by two Indium Tin Oxide (ITO) layers has most recently received particular attention due to some advantageous properties of ITO materials such as ultra-high electrical conductivity (up to 10^6^ Ω^−1^⋅m^−1^ [41]), ultra-low thermal conductivity (down to 0.84 W⋅m^−1^⋅K^−1^ [42]), superb thermal stability [43] and insusceptibility to the GST media. In this case, some preliminary study has been performed based on the previously proposed parametric approach to find out the impact of the ITO capping layer on the write process of crystalline bit, as illustrated in Figure 18. It was found in Figure 18a that for a given electrical conductivity, using a thicker ITO capping layer would remarkably reduce the temperature at certain probe points described in Figure 18b, clearly matching previous observations. This can be readily understood since a thicker ITO capping creates a more electrically resistive path between the tip and the active layer, and thereby results in lower joule heating than that with thinner ITO capping. Another crucial finding from Figure 18a is that increasing the electrical conductivity of the ITO capping layer with a fixed thickness turns out to decrease the temperature at points A–D, converse to previous reports elucidating that the temperature inside the GST layer can be heavily enhanced by increasing the electrical conductivity of the DLC capping. This discrepancy can be attributed to the large difference on the investigated electrical conductivity between ITO capping that varies from 5 × 10^−3^ Ω^−1^⋅m^−1^ to 1.25 × 10^6^ Ω^−1^⋅m^−1^ and DLC capping from 10 Ω^−1^⋅m^−1^ to 100 Ω^−1^⋅m^−1^. DLC capping exhibits a similar electrical conductivity to that of the amorphous GST and consequently the write current prefers to flow vertically through the region underneath the tip, whereas for ITO capping with an electrical conductivity much higher than the amorphous GST, majority of current would spread along the ITO capping itself other than penetrating through the Ge_2_Sb_2_Te_5_ media and therefore gives rise to lower temperature for higher electrical conductivity. As a result, the optimum values of the electrical conductivity and the thickness of the ITO capping to meet the aforementioned crystallization temperature requirements for points A-D range from 2 nm to 7 nm, and from 10^3^ Ω^−1^⋅m^−1^ to 0.5 × 10^5^ Ω^−1^⋅m^−1^ respectively, constrained in the gray region shown in Figure 18a. Notably, the 620 °C and 1400 °C contours are outside the plot, meaning that they only occur for voltage >4 V.

### 4.2. Recent Progress on Phase-Change Electrical Probe Memory from Other Groups

Encouraged by the success of inducing the phase transformation of PCMs via electrical probe, phase-change electrical probe memory has attained considerable interest during the last two decades. The first prototype of phase-change electrical probe memory was discussed in 2004 when Gidon et al. [44] designed a multilayer structure that comprised a 30 nm thick GST layer, a 2 nm DLC capping layer, and a 10 nm DLC bottom layer deposited on a Si substrate by DC-magnetron sputtering. It was reported that crystalline dots as small as 15 nm were written by a pulse of 6 V and read repeatably by a pulse of 1.7 V, demonstrating a recording density of greater than Tbit/in^2^. In 2008, Kim et al. demonstrated that electrical probes can also provide an alternative way to achieve the phase transition of GST phase-change random access memory (PCRAM) cells [45]. In their study, the recording sample had a multiplayer structure consisting of 100 nm GST film and a TiN (100 nm)/Ti (20 nm) bottom electrode, deposited on a SiO_2_/Si substrate via DC-magnetron sputtering. A two-dimensional array of GST cells with a size of 20 × 20 μm^2^ that were separated by a SiO_2_ insulation layer gap was fabricated using photoresist patterning, followed by a deposition of TiN top electrodes with a thickness of 100 nm onto each GST cell. A 10 V amorphization pulse of 50 ns and a 5V crystallization pulse of 300 ns were applied to induce the desired phase transition. Another memory structure [46], proposed by Bhaskaran et al. combined the SiO_2_ encapsulated probe mentioned in Section 3.1 with a trilayer stack consisting of a 6 nm N_2_ doped DLC capping layer, a 20 nm GST layer, and a 120 nm N_2_ doped DLC bottom layer. N_2_ doped DLC materials exhibits high electrical conductivity associated with a relatively low thermal conductivity, which should lead to a lower writing pulse and a regular-shaped bit. This speculation was verified by the resulting observation that a single crystalline bit with an average 50 nm diameter was produced by an application of a 4 V pulse of 100 μs. 

**Figure 19:**
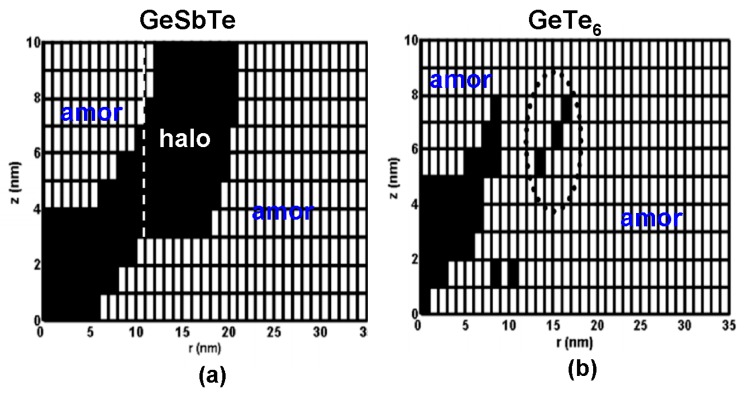
Erasing (re-amorphizing) of the crystalline bits for (**a**) conventional GST (using a 5 V, 200 ns pulse) and (**b**) a notional slow-growth material (using a 4.5 V, 200 ns pulse). Pulse rise/fall time was 20 ns in both cases. The suppression of the crystal ‘halo’ in (**b**) is clear although residues of it persist (circled). Reproduced with permission from [47]. Copyright Ieee Xplore, 2011.

In addition to the experimental research, modeling studies were also performed to determine the optimized probe memory architecture and thus help researchers better understand the physics involved into the phase transformation of PCMs for probe storage applications. Based on the model introduced in Section 3, Wright et al. [7] designed a probe memory architecture that included a Si tip and a trilayer stack comprising a 30 nm GST layer sandwiched by a 2 nm DLC capping layer with an electrical conductivity of 50 Ω^−1^⋅m^−1^ and a thermal conductivity of 100 W⋅m^−1^⋅K^−1^, and a 10 nm DLC bottom layer with an electrical conductivity of 100 Ω^−1^⋅m^−1^ and a thermal conductivity of 100 W⋅m^−1^⋅K^−1^. According to their simulations, such a structure was reported to enable a crystalline bit of 20 nm by a 10 V pulse of 100 ns, and an amorphous bit of 30 nm by a 15 V pulse of 200 ns. However, this would cause high energy consumption and is therefore not suitable for practical applications. To resolve this issue, Wright et al. has improved their previous electro-thermal model by tailoring the geometrical and electro-thermal properties of the media stack and replacing the previous JMAK equation with a newly developed nucleation-growth model [47]. Based on their report, an optimized architecture that enables ultra-high density, low power consumption, high data rate, and great rewritability, consists of a 10 nm GST layer sandwiched by 2 nm DLC capping with an electrical conductivity of 50 Ω^−1^⋅m^−1^ and a thermal conductivity of 0.5 W⋅m^−1^⋅K^−1^, and a 40 nm TiN bottom with an electrical conductivity of 5 × 10^6^ Ω^−1^⋅m^−1^ and a thermal conductivity of 12 W⋅m^−1^⋅K^−1^. Revealed by the developed nucleation-growth model, Wright et al. also found that replacing the conventional GST media with some slow crystal growth materials (such as GeTe_6_) can effectively suppress the resulting crystalline ‘halo’ formed during re-amorphization of the previously written crystalline bit, as illustrated in Figure 19. 

Later Wright’s model was expanded to three dimensions (3D) by Wang et al. [48,49,50], who also introduced more physically realistic material properties (e.g., the thermal conductivity of the DLC media) and some important electrical/thermal behaviors previously ignored by Wright (e.g., threshold switching and electrical/thermal boundary resistance) into the improved model. This resulted in a newly optimized architecture composed of a SiO_2_ encapsulated probe and a media stack consisting of 2 nm DLC capping with an electrical conductivity of 50 Ω^−1^⋅m^−1^ and a thermal conductivity of 0.5 W⋅m^−1^⋅K^−1^, a 10 nm GST layer, and a 40 nm TiN bottom with an electrical conductivity of 5 × 10^6^ Ω^−1^⋅m^−1^ and thermal conductivity of 12 W⋅m^−1^⋅K^−1^. The feasibility of using such a stack to produce ultrahigh recording density (~10 Tbit/in^2^) and ultralow energy consumption (~pJ) has already been theoretically demonstrated. 

**Figure 20:**
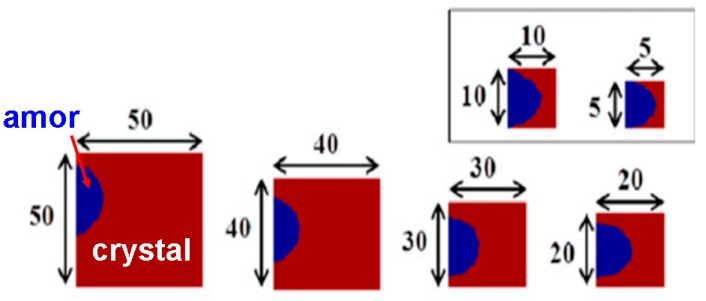
The resulting amorphous bits (blue region) embedded inside the surrounding crystalline background (brown red region) with respect to the thickness of the GST layer. The shape of the amorphous bit changes from a rounded shape for larger GST dimensions to ellipsoidal for smaller dimensions. Reproduced with permission from [51]. Copyright Ieee Xplore, 2017.

However, one severe issue of this architecture arises from the presence of excessive temperature inside the DLC capping due to its low thermal conductivity, which may melt the DLC protection layer. In this case, Wright et al. has recently proposed an alternative structure that comprise a GST layer with a thickness varying from 5 nm to 50 nm sandwiched by a 10 nm TiN capping and bottom electrode [51]. In addition, similar to [45], the GST layer in Wright’s structure is separated by a 20 nm wide SiO_2_ insulator to carefully suppress the thermal diffusion effect due to the low thermal conductivity of SiO_2_ media, thus allowing for the absence of the crystalline ‘halo’. It was clearly shown in Figure 20 that the size of the resulting amorphous bits varies from 2 nm to 10 nm by changing the thickness of the GST layer from 5 nm to 50 nm, corresponding to multi-Tbit/in^2^ areal density. More importantly, the maximum temperature induced inside the TiN capping is much lower than its melting point (~3250 K), which thus ensures the device reliability.

**Figure 21:**
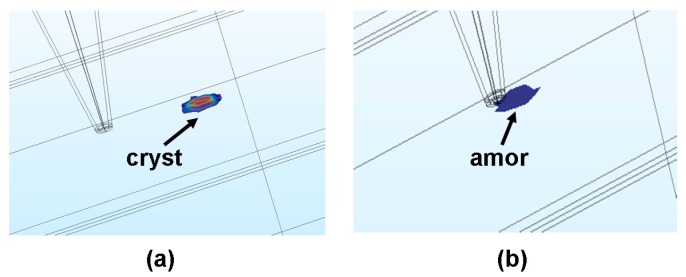
The 3D view of the resulting bits in (**a**) crystalline and (**b**) amorphous states. Reproduced with permission from [36]. Copyright Springer, 2018.

Encouraged by Wright’s findings, a parametric approach has most recently been developed by Wang et al. to calculate the maximum/minimum temperature at some pre-defined points that would intensely affect the resulting density, energy consumption [36], and device thermal stability by adjusting the geometrical and electro-thermal properties of the layered architecture including TiN capping, GST media, and TiN bottom electrode. The optimized trilayer stack is therefore established to be a 2 nm GST layer sandwiched by a 2 nm TiN capping with an electrical conductivity of 2 × 10^5^ Ω^−1^⋅m^−1^ and a thermal conductivity of 12 W⋅m^−1^⋅K^−1^, and a 40 nm TiN bottom electrode with an electrical conductivity of 2 × 10^6^ Ω^−1^⋅m^−1^ and a thermal conductivity of 12 W⋅m^−1^⋅K^−1^, deposited on Si substrate, giving rise to a cylindrical crystalline bit by a 5 V pulse of 180 ns, and a semi-elliptical amorphous bit by a 6.5 V pulse of 200 ns, as shown in Figure 21. Despite these exciting outcomes, the practicality of using designed stack to achieve the satisfying crystallization and amorhization processes still needs to be experimentally verified. 

**Figure 22:**
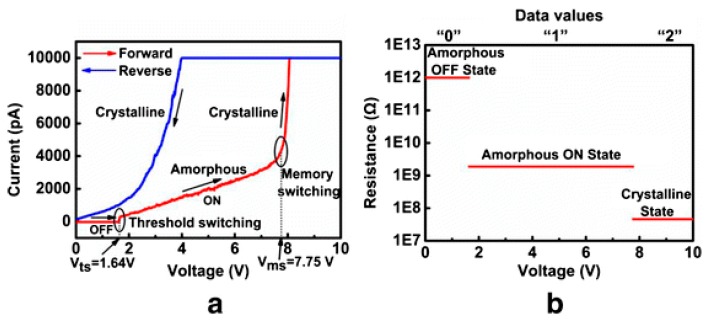
(**a**) Current-voltage spectrum of amorhous GST thin film measured in the range of 0–10 V with C-AFM. (**b**) the corresponding resistance of the amorphous OFF state, amorphous ON state and crystalline state. Reproduced with permission from [52]. Copyright Iop Science, 2015.

One of the most intriguing features of PCRAM arises from its multi-level function that was also observed in phase-change electrical probe memory by Yang et al. [51]. As reported in [52], the test sample consists of a 10 nm amorphous GST layer and a n-type Si substrate deposited on a metal sample holder via an Ag paste, while the probe tip is made of antimony doped Si coated with Pt-Ir. By changing the bias voltage applied between metal sample holder and probe tip from 0 V to 10 V, the electrical resistance of the GST layer exhibits three distinct states, namely amorphous OFF state, amorphous ON state, and crystalline state, thus indicating three storage levels (e.g., ‘0’, ‘1’, ‘2’). The corresponding I-V curves and the resulting device resistance that lead to the three different levels are schematically shown in Figure 22. 

**Figure 23:**
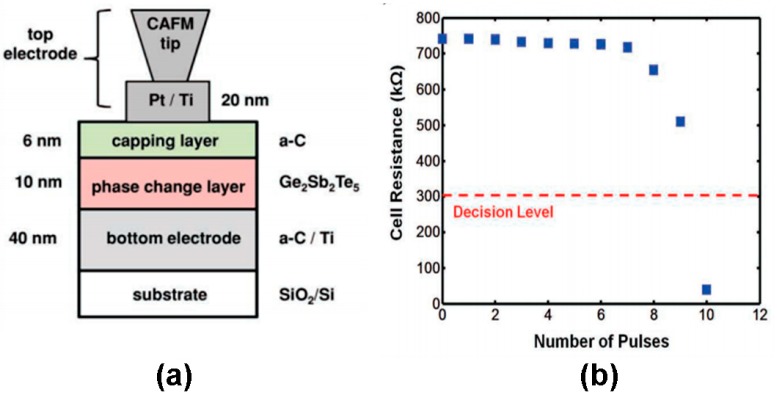
(**a**) Schematic of the phase-change electrical probe memory for arithmetic computing application and (**b**) the resistance of the cell after the application of each of 10 input pulses with each input pulse having an amplitude of 1.085 V and being of 60 ns duration. Reproduced with permission from [53]. Copyright Wiley, 2012.

Besides the storage function, phase-change electrical probe memory also exhibits an extraordinary arithmetic computing capability owing to its accumulation effects, demonstrated by Wright et al. [53]. According to Wright’s reports, such an accumulator makes use of multi-layer structure having 10 nm GST layer as the active media sandwich between a 6 nm DLC capping and a 40 nm DLC bottom electrode, while the top electrode in this case comprise a 20 nm Pt/Ti layer and a C-AFM tip. It was found that by applying successive electric pulses to the designed device, its resistance can be arithmetically divided into 10 states (named by state 1–state 10) according to appropriate adjustment of pulse magnitude and width, while the resistance at state 10 is much lower than the previous 9 states. Thanks to this, the number of the input pulses can be considered as the addends, while the complement (to the base) of the number of pulses until the cell reached its low-resistance state (i.e., state 10) will be the final outcome, which is schematically interpreted in Figure 23. Other computing such as subtraction, multiplication, and division, can be performed under the similar scenario to addition. Such arithmetic computation is carried in a non-von-Neumann architecture and thus has the potential to be extremely efficient when compared to conventional CMOS-based arithmetic processors.

**Figure 24:**
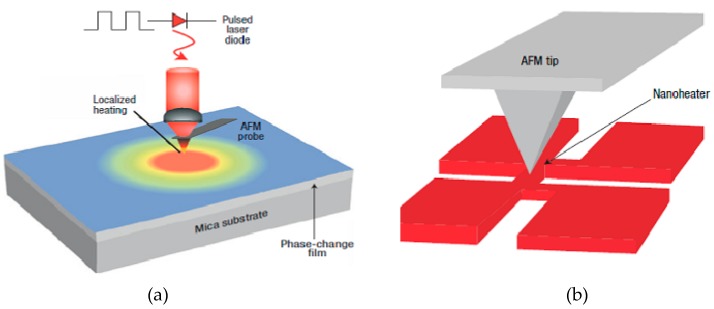
Schematic of the thermal probe storage system using phase change media when operated in (**a**) writing mode and (**b**) readout mode. Reproduced with permission from [54]. Copyright Nature, 2006.

In addition to the aforementioned technologies, another type of probe storage device that takes advantage of GST layer as the storage media and thermal-mechanical probe as the storage tool has also been reported by Hamann et al. [54], as illustrated in Figure 24. The writing operation is accomplished by bringing a thermal probe that is heated through a solid-state laser closely to the GST media where the temperature is therefore enhanced to the required temperature either for crystallization or amorphization, whereas the readout operation is performed by sensing the thermal impedance of a nanoscale resistive sensor in contact with the GST layer. This configuration is capable of detecting the phase of the GST layer directly, and may even open a way to write the crystalline bit using the heat itself. However, integrating nanoscale resistive heater with the GST media requires advanced lithography techniques, thus severely deferring the development of this technology. Table 2 summaries the aforementioned work. 

**Figure 25:**
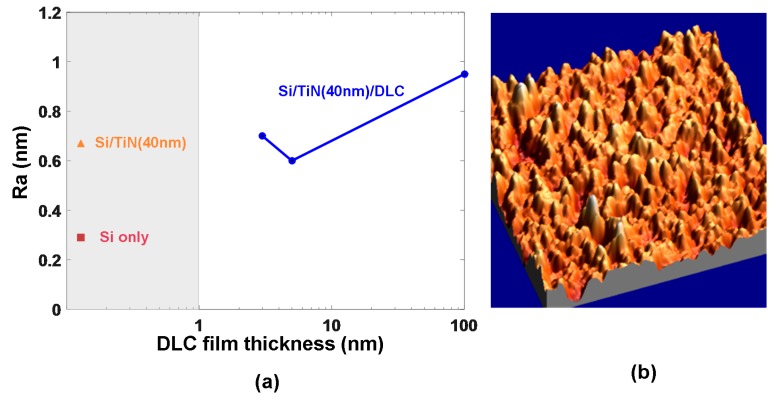
(**a**) Surface roughness obtained for different sample stacks, and (**b**) topographical image of the previously optimized sample stack with 5 nm thick DLC capping film.

According to the reported experimental observations and theoretical simulations, an optimized probe tip for phase-change electrical probe memory is required to have an electrically sharp conductive core to maintain good current conduction while having a physically blunt tip apex to effectively resist tip wear. The storage media stack is usually composed of a capping layer that protects the storage layer from wear and oxidation, a storage layer where the phase transformation takes place, and a bottom layer that acts as a bottom electrode to collect the write/read current. The design requirement for an optimized media stack is to achieve ultrahigh recording density and great reading contrast at the cost of ultralow energy consumption. To realize this, a thin capping layer with an intermediately high electrical conductivity and a low thermal conductivity is desired, while a fairly thin bottom layer with high electrical conductivity and a low thermal conductivity is usually preferable. As for the storage layer, it is necessary to have thin storage layer to reduce the entire device resistance and thus to have a bit extending through the entire layer to provide a good reading contrast. In this case, two types of the media stacks, i.e., DLC/GST/DLC, and TiN/GST/TiN, are the most commonly adopted architectures for phase-change electrical probe memory. Although vast progress on the write/read performances of phase-change electrical probe memory has recently been witnessed, it still faces some challenges that need to be overcome before it can prevail over conventional mass storage devices. Tip wear arising from the repeatable scanning process is undoubtedly the most severe issue from which phase-change electrical probe memory suffers. Despite the presence of the SiO_2_ encapsulated probe tip, the physically large tip diameter owing to the encapsulation of SiO_2_ causes fairly large forces at the tip-capping interface, thus potentially damaging the media stack. Although the force-modulation paradigm may possibly alleviate the endurance of the probe tip, load forces are needed to obtain good electrical tip-medium contact. Another challenge that phase-change electrical probe memory encounters is its inconsistency of producing storage bits of uniform size, which can be attributed to the tip wear and variation of the surface roughness distribution of the media stack [55], as illustrated in Figure 25. Most importantly, the potential applications of phase-change electrical probe memory are strongly impeded by the difficulty of amorphizing crystalline regions of the storage medium owing to the relatively slow thermodynamics of the probe tip and a high temperature present in the device that may melt the capping layer. However, it is possible to overcome this drawback by adopting TiN films as the capping media of the memory device owing to its superior stability at ultrahigh temperatures. 

Despite its various charming attributes, phase-change electrical probe memory has not yet been commercialized likely owing to its existing technological limits and the vigorous developments of Flash memory that envisage a ubiquitous application in both the industry and household fields thanks to its ultralow cost, high areal density, fast write/read speed, and long data retention. However, to reach atomic densities or atomic nanomanipulation capability, phase-change electrical probe memory is clearly the best suited and most mature technology.

## 5. Conclusions

Phase-change electrical probe memory has been unanimously considered as one of the most prospective candidates for the next-generation mass and archival storage device. To help researchers better comprehend the physics involved and to trigger more innovative technologies to further enhance its performances, the working principle of phase-change electrical probe memory associated with related experimental findings was first introduced. This was followed by a detailed discussion regarding the roles of the probe tip and media stack that included the capping layer, storage layer, and bottom layer on the write and read performances of the memory device. The amazing achievements of the phase-change electrical probe memory to date and its future possibility were discussed in conjunction with its potential weaknesses that need to be mitigated before the future commercialization occurs.

## Figures and Tables

**Table 1:** Characteristics of probe-sample device to switch PCMs.

Group	Storage Mechanism	Architecture	Bit Size (nm)	References
Kado	Electrical switch	GeSb_2_Te_4_ film Pt electrode	10–70	[16]
Tanaka	Structural switch	Ge_2_Sb_2_Te_5_ film Pt electrode	10–100	[18,19]
Pandian	Electrical & structural switch	Ge_2_Sb_2_Te_5_ film Mo electrode	~100	[20]
Sun	Electrical & structural switch	Ge_2_Sb_2_Te_5_ film Cr electrode	25–50	[21]
Maniva	Structural switch	GeTe_6_ film Ag electrode	300–1000	[22]

**Table 2:** Characteristics of phase-change electrical probe memories proposed by different groups.

Group	Architecture	Capping Property	GST Thickness (nm)	Bottom Property	Bit Image
Gidon	DLC/GST/DLC	*σ*: 10 Ω^−1^·m^−1^ L: 2 nm k: N/A	10	*σ*: 100 Ω^−1^·m^−1^ L: 10 nm k: N/A	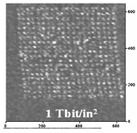
Kim	TiN/GST/TiN	*σ*: N/A L: 100 nm k: N/A	20	*σ*: N/A L: 100 nm k: N/A	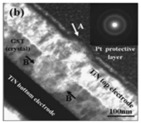
Bhaskaran	N_2_ doped DLC/GST/N_2_ doped DLC	*σ*: 166 Ω^−1^·m^−1^ L: 6 nm k: N/A	20	*σ*: 10^3^ Ω^−1^·m^−1^ L: 120 nm k: N/A	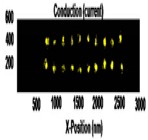
Wright	DLC/GST/DLC	*σ*: 50 Ω^−1^·m^−1^ L: 30 nm k: 100·W·m^−1^·K^−1^	30	*σ*: 100 Ω^−1^·m^−1^ L: 10 nm k:100 W·m^−1^·K^−1^	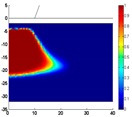
Wang	TiN/GST/DLC	*σ*: 50 Ω^−1^·m^−1^ L: 10 nm k: 0.5 W·m^−1^·K^−1^	10	*σ*: 5 × 10^6^ Ω^−1^·m^−1^ L: 40 nm k: 12 W·m^−1^·K^−1^	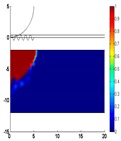
Wang	TiN/GST/TiN	*σ*: 2 × 10^5^ Ω^−1^·m^−1^ L: 2 nm k: 12 W·m^−1^·K^−1^	2	*σ*: 5 × 10^6^ Ω^−1^·m^−1^ L: 40 nm k: 12 W·m^−1^·K^−1^	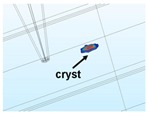

*σ*, *L*, and *k* represent electrical conductivity, thickness, and thermal conductivity, respectively.
